# Neonatal and Pediatric Organ Donation: Ethical Perspectives and Implications for Policy

**DOI:** 10.3389/fped.2015.00100

**Published:** 2015-11-17

**Authors:** Ajit A. Sarnaik

**Affiliations:** ^1^Department of Pediatrics, Wayne State University School of Medicine, Detroit, MI, USA

**Keywords:** organ donation, biomedical ethics, donation after circulatory determination of death, brain death, dead donor rule

## Abstract

The lifesaving processes of organ donation and transplantation in neonatology and pediatrics carry important ethical considerations. The medical community must balance the principles of autonomy, non-maleficence, beneficence, and justice to ensure the best interest of the potential donor and to provide equitable benefit to society. Accordingly, the US Organ Procurement and Transplantation Network (OPTN) has established procedures for the ethical allocation of organs depending on several donor-specific and recipient-specific factors. To maximize the availability of transplantable organs and opportunities for dying patients and families to donate, the US government has mandated that hospitals refer potential donors in a timely manner. Expedient investigation and diagnosis of brain death where applicable are also crucial, especially in neonates. Empowering trained individuals from organ procurement organizations to discuss organ donation with families has also increased rates of consent. Other efforts to increase organ supply include recovery from donors who die by circulatory criteria (DCDD) in addition to donation after brain death (DBD), and from neonates born with immediately lethal conditions such as anencephaly. Ethical considerations in DCDD compared to DBD include a potential conflict of interest between the dying patient and others who may benefit from the organs, and the precision of the declaration of death of the donor. Most clinicians and ethicists believe in the appropriateness of the Dead Donor Rule, which states that vital organs should only be recovered from people who have died. The medical community can maximize the interests of organ donors and recipients by observing the Dead Donor Rule and acknowledging the ethical considerations in organ donation.

## Applications of Ethical Principles to Organ Transplantation

Organ transplantation involves a potentially lifesaving gift from the donor to the recipient. However, numerous ethical considerations exist, especially in pediatrics and neonates. Practitioners involved in pediatric and neonatal organ transplantation should apply the four principles of biomedical ethics, namely autonomy, non-maleficence, beneficence, and justice (1). Autonomy is the right of self-determination. Since most children lack that capacity, we respect autonomy based on the best interest of the child as determined by a surrogate decision-maker who is usually a family member. In organ donation, the best interest of the dying child often extends to the family by bringing comfort in helping others. Non-maleficence is the principle of doing no harm. Ethically, the act of organ recovery should not cause the death of a donor, and it should not cause any pain to the donor. The logical correlate is that one should only recover vital organs from dead donors. Negatively impacting the end-of-life care of the donor could also cause harm. This can include procedures occurring before the declaration of death that benefit the recipient but offer no direct benefit to the donor, for example the consent process, procedures to preserve organ viability, and in some cases the timing and setting of withdrawal of support. Beneficence is the principle that people should do good. The medical community should give the potential donors and families the opportunity to donate because it may provide them comfort in knowing there is meaning or worth behind the death. Also, providing a potentially lifesaving organ for a recipient with end-stage organ failure is an act of beneficence. Justice is the fair and equitable allocation of resources in light of what is due to persons. One must consider whether persons dying on an organ transplant waiting list have the right to access organs of dying patients who will no longer use them.

These are *prima facie* principles in that each is binding unless it conflicts with another. For example, euthanasia and organ recovery has been performed on patients in whom the decision to withdraw support has been made (2). While we may maximize justice by providing organs to recipients who will benefit from them more than the dying patient, most in the medical community would believe that the maleficence inherent in killing a person would override that consideration. To consider the right course, practitioners must weigh all four principles of biomedical ethics, especially in pediatric and neonatal organ donation and transplantation.

## Procedures and Guidelines for Pediatric Organ Donation and Transplantation

The Organ Procurement and Transplantation Network (OPTN) is the system overseen by the US Department of Health and Human Services, which manages organ procurement, donation, and transplantation for the United States. The United Network for Organ Sharing (UNOS) is the private non-profit organization operating under OPTN that manages the organ transplantation system under contract with the federal government. UNOS manages the national transplant waiting list, maintains the database containing information on every transplant occurring in the US, and monitors organ allocation. Individual organ transplant programs and all local organ procurement organizations (OPOs) in the US are OPTN/UNOS members and are required to follow their policies. Such policies followed by transplant programs and OPOs include criteria established by the OPTN to ethically allocate organs based on many factors, including the time on the waiting list, suitability of the available organ, and benefit to the recipient. Many of these policies confer some benefit to potential pediatric recipients. For example, the new lung allocation score is based on many recipient-specific factors, such as severity of organ failure and specifics of primary illness (3). It also allows children higher priority to receive organs before they can be offered to adults. Liver allocation to children is not solely based on time on the waiting list, but also on the severity of liver disease represented by the pediatric end-stage liver disease (PELD) score or the model for end-stage liver disease (MELD) score. Revised policies assign higher priority to pediatric recipients for kidneys from donors <35 years old, and to recipients <11 years old because of the impact of renal failure on growth (4). Lastly, pediatric heart transplant candidates have additional priority over adult candidates for adolescent donor hearts (4).

In an effort to increase availability of transplantable organs, the United States Centers for Medicare and Medicaid Services mandated in 1998 that all hospitals participating in Medicare and Medicaid programs conduct timely referrals of all potential organ donors to their local OPO (5). These hospitals must also inform all families of potential organ donors of their option to donate and to allow on-site OPO personnel trained in discussing the potential for organ donation. In a 2010 policy statement that was reaffirmed in 2014, the American Academy of Pediatrics (AAP) supported of the role of the OPOs in establishing a system of approaching all potential organ donor families by individuals trained in all aspects of organ donation, including psychological, social, medical, and procedural (6). Most states are covered by more than one OPO, and every area of the country has coverage. In response to a referral from a member of the primary medical team identifying a potential donor, the OPO evaluates the potential donor, discusses donation with families, obtains consent, arranges recovery of organs, and facilitates distribution of organs according to OPTN policies.

Ethically, there should be no conflict of interest between a dying child and potential organ recipients. Perception of families and the public is also very important. Families should believe that the primary medical team and the medical community as a whole have no other consideration in mind other than the well-being of their child. If they perceive any influence of the prospect of organ donation on end-of-life decisions or recommendations, they may interpret this as a conflict of interest, even when the medical team and OPO have the best of intentions. Therefore, it is important to “decouple” the processes of declaration of death with discussion of organ transplantation. Indeed, the practices of trained OPO members approaching families and the “decoupling” process significantly increase the rate of consent for organ donation (7–10). Any member of the medical team can make a referral of a potential organ donor. Timely referral increases the chances that the family will agree to donation. A referral that occurs too late in the dying process can cause a delay in OPO personnel assessing the suitability of the potential donor, a rushed approach with the family, or donor ineligibility. The medical community’s primary responsibility is to care for the dying patient. Although there is no harm in an early referral, the prospect of organ donation must not influence medical and end-of-life decision making because it would constitute a conflict of interest. For example, withdrawal of life-sustaining therapy may be in the best interest of a child with an irreversibly devastating clinical condition that is either likely to be fatal or result in a poor quality of life. However, since these decisions are often fraught with many considerations such as clinical uncertainty, value judgments, religion, and family specific factors, it is important for the medical team to support the decision for the patient and family’s interests without considering any potential benefit to others.

## The “Dead Donor Rule”

Ethically, the removal of vital organs should not cause the death of an organ donor, because actively causing death constitutes killing. Throughout most of human existence, we have viewed death as the cessation of breathing and circulation. The medical community viewed death similarly until just a few decades ago. Indeed, the first organ transplants occurred after death was declared by circulatory criteria (11). This is known as donation after circulatory determination of death (DCDD) or non-heart-beating donation (NHBD). However, advancements in critical care made it possible to sustain circulation indefinitely in a person with no brain function. In 1968, an *ad hoc* committee from Harvard University published a report on “irreversible coma” which evolved into criteria for the neurologic determination of death, or “brain death” (12). In 1981, the US President’s Commission released the Uniform Determination of Death Act (UDDA), which states that the condition of death can be satisfied based on neurologic criteria or circulatory criteria. Brain death is the irreversible cessation of function of the entire brain, including the brainstem, and circulatory death is the irreversible cessation of circulatory and respiratory functions (13). The UDDA is a law. A person declared dead by circulatory or neurologic criteria is dead by medical, ethical, and legal standpoints. The organ transplantation community has invoked the UDDA to informally establish the “Dead Donor Rule” which states that one can only retrieve vital organs after the declaration of death. Since then, the majority of organ transplants have occurred after the donor has been declared brain dead. This is known as donation after brain death (DBD) or heart-beating donation (HBD). One benefit of DBD compared to DCDD is less ischemia and improved graft survival, because the heart is beating until the moment of organ recovery (14, 15).

The diagnosis of death is ethically and procedurally easy for a clinician if resuscitation is not undertaken. In the setting of a “do not resuscitate” order or in withdrawal of support, the clinician waits for circulation to stop, listens for heart sounds after a waiting period, and declares death. In a failed resuscitation, it can be difficult to ascertain futility of achieving return of spontaneous circulation. Diagnosing death in the context of a potential organ donation is even more challenging because one always considers the interests of the recipient to some extent while the donor is still alive. It is important for the clinician to acknowledge this fact and to minimize the conflict of interest. One method is to standardize the criteria for diagnosing death both by neurologic criteria and for circulatory criteria prior to organ donation.

## Donation after Brain Death

Although the 1968 Harvard report and the 1981 Uniform Determination of Death Act were important in laying the groundwork of diagnosing brain death, standardization was necessary to prevent variability in criteria and practice. There were several reports of children with a clinical examination consistent with brain death but who subsequently regained normal neurologic function (16). However, all of these patients were victims of accidental cold water drowning. Also, the existing criteria did not apply to children <5 years of age. In 1987, the AAP established criteria to standardize determination of brain death in children including full-term infants 7 days of age or greater (17). The guidelines emphasized determining the proximate cause of the coma to establish the irreversibility of the brain dead state. This is crucial in ruling out potentially reversible causes such as toxic and metabolic conditions, hypothermia, hypotension, and surgically remediable conditions. The presence of any of those factors precludes a diagnosis of brain death. The clinical examination consists of documenting the complete absence of function of the entire brain and brainstem. This includes complete loss of consciousness, no spontaneous movements, no reaction to stimuli, no function or reflexes of the cranial nerves, and no spontaneous breathing during apnea testing. Any evidence of brain or brainstem function precludes a diagnosis of brain death. An age-dependent period of observation between the two examinations is required to diagnose brain death. In 2011, the AAP provided an important update of the guidelines (18). Clinicians can now diagnose brain death in term newborns defined as 37 weeks gestational age or greater. The guidelines delineate the role of the ancillary studies, which include electroencephalogram (EEG) and radionuclide cerebral blood flow study. Clinicians may obtain an ancillary study if any component of brain death testing cannot be performed. Instability of respiration or circulation during apnea testing is the most common reason for this. If any component of the clinical exam is equivocal, the clinician can also obtain an ancillary study. However, they are otherwise not necessary for the diagnosis of brain death, and they do not serve as a substitute for the clinical exam. In fact, practitioners must take careful consideration prior to obtaining an ancillary study, because they cannot declare brain death if the study is equivocal or not consistent with brain death.

By the time the diagnosis of brain death is considered for a patient, the medical team should already have notified the OPO of the potential for organ donation. Ideally, OPO personnel should be on-site at that point. In fact, the US Centers for Medicare and Medicaid Services (CMS) mandates a referral of any patient with an acute brain injury with “imminent death,” which can be interpreted as a Glasgow Coma Score of <4 or 5 (19). Physicians are ethically obligated to diagnose death as soon as it has occurred, because it is in the best interest of the patient and the family. This is true under any circumstance in which death occurs, whether after withdrawal of support, an unexpected death, a failed resuscitation, or the death of a potential organ donor. Prior to death, physicians may treat a dying patient with aggressive medical management or palliative care. By diagnosing death, the medical team can shift care of the patient away from these approaches and focus either on the respectful care of a human body or the care of the organ donor, if applicable. With established brain death criteria, testing confers no conflict of interest between the patient and any potential organ recipient, because it is in the patient’s best interest to make the diagnosis. OPO personnel should initiate discussions of organ donation if the family has not already done so, because it has been shown to increase consent rates (20). The exact timing of when to approach the family is controversial. Most clinicians agree that families should not be approached before they have come to the realization that their child will die. Approaching families after the first brain death exam (if it is consistent with brain death) may balance the considerations of perceived conflict of interest and donation/transplantation logistics. At that point, the family will have had time to come to terms with the fact that the child may be already gone, and they may be receptive of information regarding donation without perceiving a conflict of interest.

If the second exam is consistent with brain death, the clinician can declare death at that time. If the child is a possible organ donor, supportive care for the organs should continue. Otherwise, care of the body should be according to the family’s wishes, unless there is a request from the medical examiner. A person who has been declared brain dead is medically, legally, and ethically dead. It is not up to a family to accept or not accept the diagnosis, and there is no consent process for disconnecting the machines maintaining breathing and circulation. Brain death can be a difficult concept for a family to process. The normative societal view of death is of a cold, gray, lifeless body. To see a loved one lying in a bed in a warm and peaceful state with vital signs on a monitor is more likely to project a view of sleep rather than death. Acknowledging this difficulty and preparing the family for death is crucial in the process of decoupling medical care from organ donation, and can help to improve consent rates.

## Donation after Circulatory Determination of Death

According to the UDDA, the other path to death besides by neurologic criteria is by circulatory criteria. DCDD is the recovery of organs for transplantation after death is declared after circulation stops followed by a waiting period in which it does not spontaneously restart. Currently, the majority of transplantable organs originate from brain dead donors. In the infancy of organ transplantation in the era prior to the establishment of brain death criteria, transplanted organs generally originated from donors declared dead by circulatory criteria (11). In the early 1990s, many institutions established protocols for DCDD in an effort to increase transplantable organs (21). Accordingly, the number of organs originating from pediatric DCDD donors has increased (22). Protocols for organ recovery in children by DCDD vary, but generally include the following (23):

- Eligibility: patients are eligible for DCDD if the decision to withdraw support has been made prior to the decision to donate organs, and if there is informed written consent for DCDD.
- Setting: withdrawal of support occurs in the operating room, perioperative area, or intensive care unit, and staff provides therapies for comfort as indicated by standard end-of-life care.
- Declaration of death: after circulation stops and does not resume after a waiting period (usually 2–5 min), a physician declares death and organs may be recovered.
- Delayed cessation of circulation: if circulation does not stop within a preset period (usually 60–120 min), the patient is no longer a DCDD candidate and end-of-life care continues.

Policy statements by the Institute of Medicine, American College of Critical Care Medicine, and the AAP have endorsed the practice and ethical robustness of DCDD (6, 24, 25). However, several studies from the perspectives of neurologists and perioperative personnel (26), pediatric critical care physicians (27), multidisciplinary task forces (28), and the general public (29) have identified ethical considerations in pediatric DCDD. Applications of ethical principles can guide the medical community in making individual and policy decisions involving DCDD.

One issue is with patient autonomy and informed consent. Children are ethically and legally incapable of providing informed consent. Although some children may have attained the developmental stage to provide assent, they are likely too ill to do so in the context of DCDD. The surrogate decision maker, who usually is a family member, provides informed consent based on the best interest of the patient. We universally consider some decisions, such as an appendectomy for appendicitis, in the best interest of the patient because it is a relatively low risk definitive cure for a condition that could otherwise be fatal. However, the only potential benefit to the DCDD donor is through the altruistic act of helping others. Some have questioned whether altruism can be presumed on behalf of a child, and therefore whether DCDD can be consistent with the best interest standard (28).

Another issue is determining the precise moment when the DCDD donor is dead and therefore eligible to undergo organ recovery. If a physician declares death inappropriately early, the death could be caused by the organ recovery. This compromises the ethical principle of non-maleficence and violates the Dead Donor Rule. If death is declared too late, a higher degree of organ ischemia occurs, compromising the ethical principles of beneficence and justice. In DCDD, a waiting period after circulation stops must be observed to ensure against the occurrence of spontaneous resumption of circulation. This phenomenon is known as autoresuscitation. There are many reports of autoresuscitation, but the vast majority occurs in adults and in the context of a failed resuscitation, which implies the presence of administered resuscitation medications in the circulation (30, 31). Some have questioned the worthiness of the Dead Donor Rule, arguing that since the declaration of death in this context is somewhat arbitrary, waiting periods can lead to the decay of the donor’s altruistic gift (32). Others have suggested revising the irreversibility criterion of the Dead Donor Rule. Although the medical team *could* restore circulation in many DCDD donors if active resuscitation is undertaken, it *would not* occur because a patient undergoing withdrawal of support does not receive resuscitation. Therefore, although circulation has not irreversibly stopped, it has permanently stopped. Accordingly, terminology of NHBD has shifted from “Donation after Cardiac Death (DCD)” to “Donation after Circulatory Determination of Death (DCDD).” Also, this distinction has led to the ethical justification of successful cardiac transplantation from a DCDD donor (33). However, some have expressed concern over changing the standard for diagnosing death in the context of organ recovery with the sole purpose of recovering more organs (34).

Providing benefit to the donor is another consideration in DCDD ethics. Many physicians believe that DCDD can benefit the donor by having a positive impact on the emotional state of the grieving family and increasing meaning and worth of the donor’s death (27). Also, focus groups at Children’s Hospital of Boston cited “making it happen for families” who wanted to help other children through the gift of organ donation as the primary reason for establishing a DCDD program (28).

Regarding benefit to society, distributive justice is defined as fair and equitable treatment in light of what is due to persons (1). People who are suffering from a disease that could be ameliorated by a transplant may have some right to receive organs that are no longer of use to their owners, i.e., dead persons and those undergoing withdrawal of life-sustaining therapies. However, the risk to the donor compels us to weigh it against other ethical principles. For example, someone who cannot consent for the procedure may suffer a violation of autonomy. Also, if medical or transplantation personnel hasten death or become influenced by a conflict of interest, a violation of non-maleficience can occur.

Overall, prominent medical societies and the literature as a whole support DCDD as an ethically sound practice and as a means to increase the supply of transplantable organs. Most practitioners support abiding by the “Dead Donor Rule.” Transplantation personnel should obtain fully informed consent with the potential donor’s best interest in mind. Physicians should optimize the exact timing of the declaration of death. Lastly, the medical community should give families who are interested in organ donation the opportunity to donate, but consider the delicate balance of approaching families without a real or perceived conflict of interest.

## Unique Neonatal Considerations in Organ Donation

Neonates represent a particularly challenging group in considering organ donation and allocation. Over the last several decades, improvements in pediatric cardiac surgery and critical care have resulted in improved corrective and palliative techniques and survival in previously lethal conditions. Similar improvements have also led to increased heart transplant recipient survival in conditions not amenable to palliation and in instances where these techniques have failed (35). While the demand for neonatal hearts for transplantation has thus increased, the gap between the supply and demand continues to grow. The mortality rate for infants <1 year of age who are waiting for a heart transplant is fivefold greater than that of the age groups of 1–5, 6–10, and 11–17 years (36).

Over the past decade, much of the research in identifying potential neonatal organ donors has centered around DCDD. In a single-center study of 117 NICU deaths over a 5-year period in babies weighing more than 2.5 kg, five infants were identified as potential DCDD heart donors because they died within 30 min of elective withdrawal of support and had good cardiac function and no active infection (37). Another single-center study of 359 deaths over a 10-year period after withdrawal of life support of neonates weighing ≥1.8 kg identified approximately 50 potential en bloc kidney donors (38). A more recent study evaluating 136 NICU deaths revealed that despite 44% meeting criteria for DCDD, fewer than 10% were appropriately referred to the regional OPO for evaluation, and only four neonates underwent successful DCDD (39). The most frequent reason for donor ineligibility was non-referral or late referral by the medical team. A study with even stricter criteria of weight threshold and warm ischemic time in a 3-year review of 200 neonatal deaths revealed the missed potential of recovering 14 livers, 18 kidneys, and 10 hearts (40). These are single-center studies each representing relatively small numbers. A concerted widespread effort to identify potential donors may lead to a higher magnitude increase of organ supply.

Interestingly, none of the above studies revealed any neonates who were declared brain dead. It is possible that brain death is rare in neonates because of the effects of open fontanels and unfused skull sutures on decreasing the likelihood of herniation and progression to brain death. However, this peculiarity could be self-fulfilling because the relative rarity of brain death in neonates may have caused a lack of awareness of the condition, leading to less brain death evaluations. It is also possible that because neonatal practitioners generally do not treat organ transplant recipients, they may have misconceptions about organ donation, lack of familiarity with DCDD and DBD processes, and inexperience in counseling families of potential donors (39).

Medical teams should refer imminent deaths to local OPOs in a timely manner. The US Centers for Medicare and Medicaid Services mandates timely referrals for organ donation for hospitals participating in the programs. There is no explicit definition of a “timely referral,” but in older children it generally means a planned or imminent death, or a neurologic injury with a GCS score of 5 or less. Clinical triggers or scoring systems for neonates have not been established, which presents an additional challenge (39).

Neonates with birth defects likely to be lethal immediately after birth represent another potential group of organ donors. Anencephaly is a severe neural tube defect in which the cerebral hemispheres including the neocortex, part of the meninges, skull, and scalp are missing. Infants born with this condition are blind, deaf, unconscious, and unable to perceive pain. Since there may be some degree of brainstem function including breathing and brainstem reflexes, it is possible for infants to live a few hours or even a few days, at which point death usually occurs. The incidence of anencephaly in the United States is approximately 1/5000 live births, which translates to approximately 700 infants born alive per year (41). Since the diagnosis is often known prior to birth, and death is certain, it is a condition that may be amenable to recovery of organs that can be perfectly viable. However, since viable organs would only originate from anencephalic infants born alive, similar concerns regarding the Dead Donor Rule and the declaration of death as in DCDD have been brought up. In the late 1980s, several ethically oriented publications pointed toward the inaccuracy in determining death in anencephalics. One argument was a potential slippery slope (42, 43). If it is justified to “use” them for their organs, people with other conditions with a poor neurologic prognosis but who are not brain dead could be similarly be “used.” At the time, other proponents of anencephalics as organ donors pointed to the marginal value of their life as humans, as never possessing even rudimentary ability to think, process sensory input, or feel pain (44, 45). In 2005, the Canadian Pediatric Society published a position paper recommending against organ donation from anencephalic neonates due to the difficulty in establishing brain death in this condition (46). The statement also cites the risks of the “slippery slope” in using organs from other brain-damaged living people, risk of losing public trust in transplantation programs, and risk of losing public respect for value of human life. Since then, there have been relatively fewer reports in the literature regarding anencephalic organ donation. However, as neonatal organ donation is becoming recognized as a potential bridge to the gap between supply and demand for transplantable organs, it could become more relevant. Wijetunga et al. reported the organ recovery of an anencephalic infant in 2015 (47). This case involved a twin gestation, and the condition of the affected fetus was known prenatally. The parents were counseled prior to the baby’s birth and agreed to organ donation. The kidneys were accepted for potential transplant and the organ recovery team was mobilized when the mother was in labor. The baby was born alive, and had circulatory arrest and organ recovery 1 h, 35 min after delivery. The ethical considerations in this case, including declaration of death and potential conflict of interest, are similar to that in DCDD because the procedure of organ recovery was in fact DCDD. It was not clear in this case whether the parents initiated the discussion of organ donation prenatally, or if it was the primary medical team or OPO. Regarding DCDD, any discussion of organ donation occurring before death can present a conflict of interest between the medical community and the potential donor. Neurologic prognosis for a living patient could be affected by a potential gain to the medical community or transplant recipients. Even if that is not the case, families could perceive it, which could lead to mistrust. However, this issue is less relevant in anencephalic organ donation compared to other disease states because the prognosis and the complete lack of meaningful neurologic function are more certain. This case establishes the procedural and ethical feasibility of organ recovery from anencephalic neonates, which can be accomplished as long as uniform criteria for death are observed and ethical considerations are acknowledged.

## Conclusion

The overarching goal for neonatal and pediatric organ donation and transplantation is to maximize benefits to the donors and the recipients without causing harm or violating autonomy. The medical community can approach this goal by providing families of dying children the opportunity to donate without an actual or perceived conflict of interest between potential donors and recipients. One can minimize harm to the donor with application of firm criteria for neurologic or circulatory criteria prior to organ recovery. Systems are in place to ensure timely referrals of potential donors, to empower trained individuals of OPOs to discuss organ donation with families, and to appropriately allocate organs based on donor-specific and recipient-specific factors.

## Author Contributions

AS, M.D., was responsible for the concept, literature review, and manuscript preparation.

## Conflict of Interest Statement

The author declares that the research was conducted in the absence of any commercial or financial relationships that could be construed as a potential conflict of interest.
